# A dataset of perception and preferences of French consumers for commercial cooked hams sampled according to their nutritional values and claims.

**DOI:** 10.1016/j.dib.2024.110549

**Published:** 2024-05-23

**Authors:** Michel Visalli, Anne-Laure Loiseau, Sylvie Cordelle, Benjamin Mahieu, Pascal Schlich

**Affiliations:** a Centre des Sciences du Goût et de l'Alimentation, AgroSup Dijon, CNRS, INRAE, Université Bourgogne, F-21000 Dijon, France; b INRAE, PROBE research infrastructure, ChemoSens facility, F-21000 Dijon, France; c Oniris, INRAE, StatSC, 44300 Nantes, France

**Keywords:** Sensory evaluation, Consumer choices, Field experiment, Free comment, Just-about-right scales, Hedonic test

## Abstract

This article describes a dataset providing sensory and nutritional information for 30 commercial cooked hams (without rind, not flavored) representative of the French commercial segment.

The sensory data were collected in two phases. During the first phase (fall 2019, field experiment), 483 consumers, regular consumers of cooked hams, were recruited in seven cities and vicinities of France. They were instructed to choose and buy cooked hams at the supermarket and evaluate them at home over a period of three months. They were provided with a list of 30 eligible cooked hams selected by the experimenters. A total of 2758 evaluations were collected (an average of 5.7 evaluations per consumer). During the second phase (fall 2020, lab experiment), a selection of 16 cooked hams were evaluated at blind by 86 consumers in a sensory analysis laboratory using a complete balanced design. Sensory evaluation at home and in the laboratory included liking Just-About-Right (colour, fat, salt and texture) measurements. In the field experiment, consumers were additionally asked to describe with free comments the appearance, texture and flavour of tested hams and of a virtual “ideal ham”. They also had to report the price they paid for a pack of four slices of ham and their intentions to repurchase the tested hams. Other data on cooked hams included actual salt and fat contents (measured using physicochemical analyses) and information displayed on the packaging (type of brand, nutritional claims, labels).

This dataset offers a broad overview of the perception and the appreciation of cooked hams representative of the French market, and it could allow the joint analysis of intrinsic and extrinsic food properties. Moreover, this data paper describes an innovative protocol of field experiment bridging the gap between the controlled lab environment (panelized consumers, selection of the list of hams by the experimenter) and the real-life settings (hams chosen by the consumers and tasted at home with access to information). Such a protocol could be reused to collect large sensory datasets and aggregate them into open databases interoperable with other food databases (nutritional, economic, sustainability, etc.).

Specifications Table

**Table:** 

Subject	Food science
Specific subject area	Sensory evaluation
Type of data	Table, Figure Raw
Data collection	For sensory evaluation, 483 consumers (field experiment) and 86 consumers (laboratory experiment) were asked to evaluate commercialized cooked hams using TimeSens [1] software. They had to report their liking scores and sensory descriptions of appearance, texture and flavour of the products using the free comment method). They also had to describe sensory aspects of their ideal cooked ham. Extrinsic information displayed on the packaging of cooked hams were collected and reported by the experimenters. Analyses of nutrient composition were performed in an extern laboratory (Eurofins Analytics France, NF EN ISO/IEC 17025:2005 COFRAC 1-0287 accreditation). Total fat composition was measured by gravimetry (microwave). The method is based on the hydrolysis and the extraction of fat by sulphuric acid and cyclohexane. Total fat is determined by gravimetry after evaporation of cyclohexane. Sodium was measured by Flame Atomic Absorption Spectrometry. The amount of sodium is dosed after wet mineralisation by spectrometry of atomic absorption.
Data source location	Consumers were recruited in seven cities and vicinities by the following partners. The data were sent from consumer homes to CSGA (Dijon) directly by internet. The laboratory test data were collected in the ChemoSens sensory booths in Dijon. Salt and fat compositions were analyzed at EUROFINS Laboratoire de microbiologie ES, 16 rue Clément Ader, 68127 Sainte Croix en Plaine, France.
Data accessibility	Repository name: Recherche Data Gouv Data identification number: 10.57745/GA5R6S Direct URL to data: https://doi.org/10.57745/GA5R6S [2]
Related research article	B. Mahieu, M. Visalli, P. Schlich. Identifying drivers of liking and characterizing the ideal product thanks to Free-Comment, Food Quality and Preference (2022), 96, 104389, https://doi.org/10.1016/j.foodqual.2021.104389 [3]

## 1 Value of the Data

• These data are unique and useful because they offer a broad overview of the sensory perception and appreciation of a representative sample of the commercial segment of cooked hams distributed in French supermarkets.
• The protocol of data collection at home is innovative: the field experiment bridges the gap between the controlled lab environment (panelized consumers, selection of the list of hams by the experimenter) and the real-life settings (hams chosen by the consumers and tasted at home with access to information). Such a protocol could be reused to collect large sensory datasets and aggregate them into of open databases interoperable with other food databases (nutritional, economic, sustainability, etc.).
• Data were also collected in the laboratory with hams tasted at blind, allowing comparison of the two contexts of sensory evaluation.
• Any researcher interested in relating sensory attributes, nutrient composition or purchase context of cooked hams to their sensory acceptability by consumers can benefit from these data. Indeed, they can help to better understand consumer choices based on intrinsic and extrinsic information, and to assess the hedonic cost of the fat and salt reductions made necessary for public health reasons.

## 2 Background

In France, there are several open access databases on the nutritional or environmental quality of foods, but there is nothing of the sort on their sensory qualities and their levels of appreciation by consumers. As sensory aspects remain the first driver of re-purchasing, it is of paramount importance to build sensory perception food databases in order to relate reformulate healthier and more sustainable food products.

Due to the large numbers of references on the market, collecting sensory description and liking data by classical tasting in laboratory is just impossible. To overcome this difficulty, we designed an innovative field experiment protocol that allows consumers to evaluate the products they purchase at home using any device (smartphone, tablet or PC). The original research article focused on the identification of drivers of liking and the characterization of the “ideal cooked ham”, but there is much more information available in this dataset.

## 3 Data Description

The dataset is in a form on an XLXS file, with 8 datasheets. Missing data in cells is referred to as “NA”.

**Tab “description”**

Correspondences between variable names by datasheet and their types and description.

**Tab “product packaging”**

Information about hams, as displayed on the packaging in date of 2019-08-26.

- *Product* is the 3-digit anonymized unique identifier of the ham.
- *BrandType* is the brand type of the product (Retailer or National brand).
- *CompanyCode* is an anonymized code for the company having produced or made produced the ham (NBi for National brand I and Rj for Retailer j)
- *Organic, NoNitrite, SaltLight, FatLight, LabelRouge* take the values 1 if the ham is respectively organic, has no nitrite, is light in salt (−25%), is light in fat, is certified with Label Rouge.

**Tab “product composition”**

Fat and salt contents were measured at three different dates: 2019-10-29, 2019-12-04 and 2019-12-13.

- *Product* is the 3-digit anonymized unique identifier of the ham.
- *Replicate* is the replicate number of the analysis (batch).
- *ExpirationDate* is the expiration date of the ham.
- *FatMeasured, SaltMeasured* are measured fat and salt contents (g for 100 g).

**Tab “consumer”**

Information about the consumers, collected during the recruitment phase (at home and in the laboratory):

- *TestLocation* is the location of the test (home or lab).
- *Consumer* is the unique identifier of the consumer, prefixed by H (home) or L (lab).
- *DateStart* is the start date of the data collection by the consumer.
- *DateEnd* is the end date of the data collection by the consumer.
- *City* is the code of the city and vicinity where the consumer was recruited (1: Bourg-en-Bresse, 2: Caen, 3: Strasbourg, 4: Agen, 5: La Rochelle, 6: Angers, 8: Dijon), 9: Dijon (laboratory experiment).
- *Gender* is the gender of the consumer (M for male or F for female).
- *Age* is the age range of the consumer (18–30, 31–50, 51+).
- *ConsumptionFrequencyByMonth* is the answer given to the question “What is your usual consumption of cooked ham?”, recoded as 1 (“Less often than once every 2 weeks”), 2 (“About once every two weeks”), 6 (“Once or twice a week”) or 12 (“Three times a week or more”).

Note: for data collected in lab, DateStart and DateEnd were the same because all products were tasted on the same day.

**Tab “consumer questionnaire (home)”**

Raw data related to the consumers collected from the online survey using TimeSens software (field experiment only):

- *Consumer* is the unique identifier of the consumer.
- *DifficultyFreeComment* is the answer given to the question “Did you find it difficult to describe the ham in free text” recoded as -2 (“Very difficult”), -1 (“Difficult”), 0 (“Neither easy nor difficult”), 1 (“Easy”), 2 (“Very easy”).
- *IdealVisual* is the answer given to the question “Could you describe the visual aspect of an ideal ham in your opinion?” (free comment , in French).
- *IdealFlavor* is the answer given to the question “Could you describe the taste of an ideal ham in your opinion?” (free comment, in French).
- *IdealTexture* is the answer given to the question “Could you describe the texture of an ideal ham in your opinion?” (free comment, in French).

**Tab “consumer questionnaire (lab)”**

Raw data related to the consumers collected from the online survey using TimeSens software (laboratory experiment only):

- *Consumer* is the unique identifier of the consumer.
- *ZeroNitrite* is the answer given on a Likert scale to the question “To what extent do you agree with the assertion: I make sure to buy nitrite free ham?”, recoded as -2 (“Fully disagree”), -1 (“Disagree”), 0 (“Neither agree nor disagree”), 1 (“Agree”), 2 (“Fully agree”).
- *Organic* is the answer given to the question “To what extent do you agree with the assertion: I make sure to buy organic ham?”, recoded as -2 (“Fully disagree”), -1 (“Disagree”), 0 (“Neither agree nor disagree”), 1 (“Agree”), 2 (“Fully agree”).
- *LabelRouge* is the answer given on a Likert scale to the question “To what extent do you agree with the assertion: I make sure to buy ham with a quality label (e.g.: label rouge)?”, recoded as -2 (“Fully disagree”), -1 (“Disagree”), 0 (“Neither agree nor disagree”), 1 (“Agree”), 2 (“Fully agree”).
- *LessFat* is the answer given on a Likert scale to the question “To what extent do you agree with the assertion I make sure to buy low fat ham?”, recoded as -2 (“Fully disagree”), -1 (“Disagree”), 0 (“Neither agree nor disagree”), 1 (“Agree”), 2 (“Fully agree”).
- *LessSalt* is the answer given on a Likert scale to the question “To what extent do you agree with the assertion: I make sure to buy salt reduced ham?”, recoded as -2 (“Fully disagree”), -1 (“Disagree”), 0 (“Neither agree nor disagree”), 1 (“Agree”), 2 (“Fully agree”).
- *TypeBrand* is the answer given to the question “What type of cooked ham brands do you consume most often”: “National brand”, “Retailer”, “Discount” or “SoldByTheCut”.

**Tab “product sensory properties”**

Raw data related to the products’ sensory properties collected from the online survey using TimeSens software (field and/or laboratory experiment):

- *Consumer* is the unique identifier of the consumer.
- *Product* is the 3-digit anonymized unique identifier of the ham.
- *Liking* is the liking score given on a 0-10 VAS scale.
- *JARColor, JARFat, JARSalt, JARTender* are the answers given to the “Just About Right” questions related to colour, fat, salt and tender “According to you, this ham is:” recoded as -2 (“Really not enough…”), -1 (“Not enough”), 0 (“Just about right”), 1 (“Too much…”), 2 (“Really too much…”).
- *DescriptionVisual* is the answer given to the question “Please describe the visual aspect of this ham” (free comment, in French, at home only).
- *DescriptionTexture* is the free text answer given to the question “Please describe the texture in the mouth of this ham:” (free comment, in French, at home only).
- *DescriptionFlavor* is the free text answer given to the question “Please describe the taste of this ham” (free comment, in French, at home only).

**Tab “product purchase information (home)”**

Raw data related to the products’ purchase information collected from the online survey using TimeSens software (field experiment only):

- *Consumer* is the unique identifier of the consumer.
- *Product* is the 3-digit anonymized unique identifier of the ham.
- *HamUsuallyBought* is the answer given to the question “Are you used to buy or consume this ham (same brand and same reference)?” recoded as 1 (“Yes”) or 0 (“No”).
- *PurchaseIntent* is the answer given to the question “Would you buy this product again?” recoded as -1 (“No”), 0 (“I don't know”) or 1.
- *Price* is the price in euros of the ham at the moment of the purchase, as reported by the consumer.

## 4 Experimental Design, Materials and Methods

### 4.1 Overview

The objective of this study was to collect sensory perception data representative of ham sold in supermarkets in order to aggregate them in a database and link them to nutritional data to study a potential reduction in fat or salt. Data were collected in two phases. During the first phase (field experiment, fall 2019), sensory data of 30 cooked hams were collected at home using an internet questionnaire. A total 483 panelised consumers were recruited for this purpose. During the second phase (laboratory experiment, fall 2020), a subset of 16 cooked hams was evaluated by 86 consumers in a sensory laboratory.

### 4.2 Phase 1: field experiment

#### 4.2.1 Ham selection

**Fig. 1:**
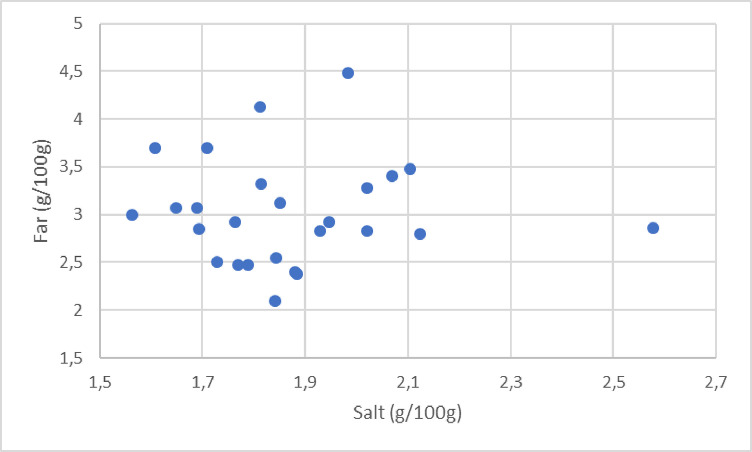
Mean salt and fat contents of the cooked hams.

In this study, it was decided to restrain to cooked hams without rind. The references were selected using the Open Food Facts database [4]. Open Food Facts is a large database of food products with ingredients, allergens, nutrition facts and information found on product labels. Smoked, braised, spit-roasted, flavoured hams and hams with rind were excluded. A total of 288 references were retrieved. As the hams had to be purchased by consumers and evaluated at home, only hams presented in packages of four slices were kept. Out-of-date products (recipe, packaging) were not retained, as well as those that were not available in the supermarkets of the cities in which the study took place, which restricted the selection to 74 products. Finally, a sample of 32 hams was selected so as to be as balanced as possible in terms of fat and salt levels (Fig. 1) and types of brands (16 national brand references, 16 from resellers). Different types of claims were present on the ham packaging (two organic ham references, three nitrite-free, four low-salt, one low-fat, one Label Rouge certified). Two hams were withdrawn from the market at the beginning of the study. Thus, only 30 hams were evaluated.

#### 4.2.2 Ham composition

During the evaluation period, four to seven different batches of each ham were purchased and stored in CSGA'S refrigerated equipment. Analyses of composition were carried out in an external laboratory: Eurofins Analytics France (NF EN ISO/IEC 17025:2005 COFRAC 1-0287 accreditation). Technical staff from the laboratory took care the transport of the samples (refrigerated transport). One or two ham per batch were used to prepare the sample according to the standard NF V04-416. Fat and salt contents were analysed. Total fat composition was measured by gravimetry (microwave). The method is based on the hydrolysis and the extraction of fat by sulphuric acid and cyclohexane. Total fat is determined by gravimetry after evaporation of cyclohexane. Sodium was measured by Flame Atomic Absorption Spectrometry. The amount of sodium is dosed after wet mineralisation by spectrometry of atomic absorption. Salt content is calculated using the following formula: salt = sodium x 2.5.

#### 4.2.3 Other information about ham

The information displayed on the packaging of each ham (brand type, claims and labels) was collected and reported by the experimenters.

#### 4.2.4 Participants

Recruitment and remuneration management was supervised by partners from seven sensory analysis laboratories in different regions of France (cities: Agen, Angers, Bourg-en-Bresse, Caen, Dijon, La Rochelle, Strasbourg). A total of 538 panelised consumers were recruited. They had to be regular consumers of cooked ham, at least once every two weeks (mean = 6.6 times a month). They were informed that they had to purchase and evaluate at home a minimum of four different hams and a maximum of different 12 hams among a predefined selection of 32 hams. Each participant was informed of the conditions for participating and signed a consent form. A total of 483 consumers finally participated. The panel was slightly unbalanced, with an over-representation of women and 31–50 year olds (Table 1). They received compensation at the end of the study in the form of a voucher, the amount depending on the numbers of evaluated hams: € 10 for four or five hams, € 15 for six and seven hams, € 20 for eight and more hams. The study lasted approximately three months.

**Table 1:** Field experiment panel composition, by age, gender, and laboratory.

Sensory lab	1	2	3	4	5	6	8	Total
F	**58%**	**58%**	**56%**	**60%**	**64%**	**56%**	**56%**	**58%**
18–30	5%	20%	14%	15%	8%	13%	19%	14%
31–50	33%	25%	22%	29%	34%	25%	15%	25%
51+	19%	13%	19%	16%	22%	18%	22%	19%
M	**42%**	**42%**	**44%**	**40%**	**36%**	**44%**	**44%**	**42%**
18–30	7%	3%	4%	3%	0%	7%	7%	5%
31–50	21%	18%	26%	18%	15%	20%	29%	22%
51+	14%	20%	14%	19%	20%	18%	8%	16%
Total	**100%**	**100%**	**100%**	**100%**	**100%**	**100%**	**100%**	**100%**

#### 4.2.5 Protocol

Each partner sent an email to its consumers inviting them to connect to TimeSens, a web application dedicated to the collection of sensory data. Once logged in, the consumer had to confirm having read the instructions attached to the email. If he did not confirm (screen 2), he was invited to read them before to continue (screen 3). Then, he had to answer two control questions: “How many hams do you have to eat at least to get compensated?” (screen 4) and “If you evaluate 10 hams in this study, how much will you be compensated?” (screen 5). These questions were asked only once, to check if the consumer read the instructions.

Then, a reminder of the conditions of the study was shown on three successive screens (screens 6-8). The first screen (screen 6) displayed the following text: “This study will take place over eight weeks. As part of this experience, the amount of compensation will be: 10 euros for four or five hams evaluated, 15 euros for six or seven hams evaluated, 20 euros for eight or more hams evaluated. The evaluation of the hams will always be done with the same internet link. All you have to do is reconnect using it to evaluate a new ham. You must respect a minimum of 24 h between each ham evaluated.”. The second screen (screen 7) displayed the following text: “Before each product review, we will ask you to take a photo of the product. This will constitute proof of purchase. As a reminder, the ham must be tasted alone without any other accompaniment (without bread, without butter, without anything else). When evaluating the ham, you will need to describe: its visual appearance, its texture in the mouth, its taste. Finally, you will give your appreciation of the ham tasted.”. The third screen (screen 8) displayed the following text: “You will have to describe your perception of the product (visual appearance, texture, taste) with your own words. Fictitious example of the description of the taste of an orange: *This orange is bloody, a little acidic, very fruity and reminds me a little of my childhood*. You can use terms of texture, appearance, flavours, aromas, etc. as well as emotions or any other sensation. You can also use quantifications (very, a little, a lot, etc.). There is no limit to the length of your answer.”.

The consumer had two options (screen 9): select the identifier (EAN) of the ham just purchased in a dropdown list, or declare he no longer wants to evaluate hams. To ensure he really bought the product, he first had to take a picture of the package, after opening (screen 10). Then, he had to score his hedonic appreciation on a 0-10 VAS scale (screen 11): “How do you like this ham?”.

Then, questions about the sensory perception of the ham were asked using the method of free comment by modality: “Please describe the visual aspect of this ham” (screen 12), “Please describe the texture in the mouth of this ham” (screen 13), “Please describe the taste of this ham” (screen 14).

Then, 5-point Just About Right (JAR) scales were presented (screens 15-18), asking the consumer: “According to you, this ham is:…”. The left anchor was “Really not enough bright/salty/fatty/tender, the right anchor was “Really too much dark/salty/fatty/tender”, and the medium anchor was “Just about right”.

After having described his sensory perception, the consumer should fill in a questionnaire about his purchase habits. The following questions were asked: “Do you usually buy or consume this ham (same brand and same reference)?” (screen 19); “Would you buy again this product?” (screen 20); “What is the price (in euros) of the ham at the moment of the purchase” (screen 21).

Finally, a reminder screen was displayed (screen 22): “To validate the evaluation of the ham that you have just made, click below by selecting either CHOICE 1 or CHOICE 2. Attention! By clicking on CHOICE 2, your participation in this study will be definitively finished and you will no longer be able to evaluate other hams. Reminder: you must evaluate at least four hams to obtain a compensation.”. If the consumer clicked on the button corresponding to “CHOICE 1”, he had to reconnect on a different day and to repeat the procedure from the entering of the EAN.

If the consumer clicked on the button corresponding to “CHOICE 2”, a final questionnaire had to be completed before being eligible to compensation. On three different screens, free comment questions were asked about a potential “ideal ham” as follows: “Could you describe the visual aspect of an ideal ham in your opinion?” (screen 23), “Could you describe the texture of an ideal ham in your opinion?” (screen 24), “Could you describe the taste of an ideal ham in your opinion?” (screen 25). Then, the following questions were asked (screen 26): “Did you find it difficult to describe the ham in free text” (5-points scale: “Very difficult”, “Difficult”, “Neither easy nor difficult”, “Easy”, “Very easy”). Finally, the following questions were asked (screen 27): “What is your usual consumption of white ham?”, “What is your gender?”, “How old are you?”.

A total of 415 consumers remained in the study until the final questionnaire.

### 4.3 Phase 2: laboratory Experiment

#### 4.3.1 Ham selection

The second phase was set up to compare the results obtained at home with the results (with information) obtained in the laboratory (in blind conditions). For feasibility reasons, a subset of 16 hams among the 30 remaining products was selected. They were chosen to be as balanced as possible in terms of fat and salt levels, types of brands, and hedonic scores based on the field experiment (Table 2).

**Table 2:** Hams selected for the laboratory experiment, based on the results of the field experiment.

Product	Measured fat (g/100g)	Measured salt (g/100g)	Organic	Low-salt	Nitrite-free	Low-fat	Label rouge	Average Price (€)	Number of evaluations (2019)	Mean hedonic score (2019)
J1	2.63	1.44		X				2.43	263	6.49
J2	2.48	1.79					X	2.95	192	6.97
J7	4.48	1.98	X					4.45	42	5.49
J9	2.80	2.12			X			2.86	112	7.44
J10	2.10	1.84						2.69	146	6.94
J11	1.85	1.44		X		X		3.00	42	5.77
J16	3.48	2.11						1.58	25	3.36
J21	2.93	1.76					X	2.71	69	5.99
J22	3.70	1.61	X					4.82	34	6.44
J23	2.40	1.88			X			2.10	101	5.70
J24	2.55	1.84						2.82	61	6.61
J25	4.13	1.81						1.64	44	4.74
J26	2.50	1.73						2.25	200	6.41
J27	2.85	1.69						2.65	136	6.63
J30	2.48	1.77			X			2.64	172	6.64
J31	3.63	1.47		X				2.46	175	6.77

#### 4.3.2 Participants

A total of 86 panelised consumers, regular eaters of cooked ham (mean = 6.1 times a month) were recruited from a population registered in the ChemoSens Platform's PanelSens database. This database has been declared to the relevant authority (Commission Nationale Informatique et Libertés—CNIL—n° d'autorisation 1148039). The panel was almost balanced in terms of age repartition and gender (Table 3).

**Table 3:** Home experiment panel composition, by age and gender.

	F	M	Total
18–30	9 (22.5%)	11 (23.9%)	20
31–50	13 (32.5%)	19 (41.3%)	32
51–70	18 (45%)	16 (34.8%)	34
Total	**40**	**46**	**86**

Each participant was informed of the conditions for participating and signed a consent form. They received compensation at the end of the study in the form of a voucher of € 10.

#### 4.3.3 Protocol

All 16 hams were evaluated blindly in one session at 11:00 or 12:45 in individual sensory booths. The products were presented according to a William's Latin square design. The samples were served under white light at a temperature of around 4° C (tasting immediately out of the refrigerator, no waiting at room temperature), quarter-sliced ​​in a small plastic plate. The consumers could consume as much ham as they wanted. Before tasting each ham, they had to rinse their mouths with water. A 10-minute mid-session break was forced. The session lasted for about one hour.

The data were collected with TimeSens software following this protocol. General instructions were presented on the first screen as follows: “You will receive 16 pieces of ham, one after the other. We ask you to taste each ham. Then you will indicate how much you like it, as well as your perception of its colour, its saltiness, its fatness and its tenderness. Each portion served represents 1/4 of a slice of ham (in total, you will receive the equivalent of four slices of ham). You can eat as much as you want (you don't have to finish the portions).”. Then, they had to score their liking and their JAR perception with the same procedure as in the field experiment.

Once the 16 hams were tasted, a questionnaire had to be completed, including the following questions: “What type of cooked ham brands do you consume most often”; “To what extent do you agree with the assertions” (“Fully disagree”, “Disagree”, “Neither agree nor disagree”, “Agree”, “Fully agree”): “I make sure to buy nitrite free ham”, “I make sure to buy organic ham”, “I make sure to buy ham with a quality label, e.g., label rouge”, “I make sure to buy low fat ham”, “I make sure to buy salt reduced ham”.

## Limitations

Free comment descriptions are in French.

## Ethics Statement

As the objective of the study was to evaluate the sensory properties and preferences for commercially available food products, ethical approval by an institutional research board was optional to conduct the research (French law n°2012-300 of March 5, 2012 relating to Research Involving Human Persons). The study received approval from the local ethics committee of the research center. Each participant was informed of the conditions for participating and signed a consent form. The research was carried out in conformity with the Declaration of Helsinki.

## CRediT authorship contribution statement

**Michel Visalli:** Formal analysis, Software, Data curation, Writing – original draft. **Anne-Laure Loiseau:** Methodology, Resources, Validation, Project administration, Investigation, Data curation. **Sylvie Cordelle:** Methodology, Resources, Validation, Project administration, Data curation. **Benjamin Mahieu:** Formal analysis, Validation. **Pascal Schlich:** Conceptualization, Methodology, Formal analysis, Supervision, Funding acquisition.

## Data Availability

A dataset of perception and preferences of French consumers for commercial cooked hams sampled according to their nutritional values and claims. (Original data) (Recherche Data Gouv).
